# Overlap and Segregation among Multiple 3D Home Ranges: A Non-Pairwise Metric with Demonstrative Application to the Lesser Kestrel *Falco naumanni*

**DOI:** 10.3390/biology12010077

**Published:** 2023-01-02

**Authors:** Alessandro Ferrarini, Giuseppe Giglio, Stefania Caterina Pellegrino, Marco Gustin

**Affiliations:** Lipu-BirdLife Italy, Via Udine 3/a, I-43122 Parma, Italy

**Keywords:** 3D home range, 3D overlap/segregation estimator, animal space overlap, animal space use, biotelemetry, height above ground level, tessellation, topographic surface

## Abstract

**Simple Summary:**

Modeling animal space use in 3D is more realistic than confining research to 2D methods and can greatly increase our understanding of interspecific and intraspecific competition, predator–prey relationships, habitat selection and use. In particular, home range overlap/segregation is a fundamental property of animal interactions with deep implications for biodiversity conservation and management. In order to solve the issue of measuring the degree of overlap/segregation among an arbitrarily large number of 3D volumetric home ranges, we introduced the novel non-pairwise index *MVOI* (Multiple Volumetric Overlap Index) and its complement to 100 *MVSI* (Multiple Volumetric Segregation Index). Results show that traditional 2D spatial analyses can significantly overestimate the overlap between the individuals, population and species that occupy a habitat with a strong vertical component. Both the *MVOI* and *MVSI* can also be used to quantify how the 3D home ranges change over time (i.e., 4D home ranges) and the robustness of 3D home range assessment through the degree of overlap among different 3D estimators. We applied the *MVOI* and *MVSI* to birds, but they can be readily applied to any animal species, in particular those with a significant vertical component to their space use.

**Abstract:**

In this study we solved the issue of measuring the degree of overlap/segregation among an arbitrarily large number (*n* ≥ 2) of 3D volumetric home ranges (i.e., *x*, *y*, and *h_g_*; where *h_g_* is height above ground level) for the first time. For this purpose, we introduced the novel non-pairwise index *MVOI* (Multiple Volumetric Overlap Index) and its complement to 100 *MVSI* (Multiple Volumetric Segregation Index). Regardless of the number of 3D volumetric home ranges, the *MVOI* and *MVSI* generate a single score of overlap/segregation between 0 and 100, making ecological interpretation much easier and more meaningful when compared to *n* × *n* pairwise overlap indices. As a case study, we applied the *MVOI* and *MVSI* to 12,081 GPS points of five lesser kestrels (*Falco naumanni*) during the nesting period at Santeramo in Colle (Apulia region; Italy) in an area with the most elevated density of lesser kestrels in urban colonies worldwide. The 3D volumetric home ranges ranged between 1.79 km^3^ and 8.19 km^3^. We found that the tracked birds had different vertical profiles, possibly to limit intraspecific competition, resulting in a 3D home range overlap that was only 61.1% of the 2D overlap and 52.8% of the probabilistic one.

## 1. Introduction

Space use by animals can reveal many important ecological processes, such as foraging requirements [1], intra- and interspecific competition [2], habitat use and selection [3], territoriality [4], the key factors affecting movement [5] and their fitness [6].

One main measure of space use is the home range (HR hereafter), i.e., the space used by an individual to obtain resources and meet its requirements for survival and reproduction [7]. Although the HR has been described for many taxa almost exclusively in two dimensions (*x*, *y*), for species that have a strong vertical component (*z*) to their movement (i.e., height above ground level for flying species, depth for fish species and elevation for species moving in highly variable terrain), such two-dimensional (2D) representation neglects some key components of their ecology, such as the actual size of their HRs and overlap between the HRs. Several authors [8,9] showed that a 2D representation can underestimate the surface area of HRs relative to three-dimensional (3D) estimates for animals that occupy habitat with a strong vertical component. The interspecific and intraspecific vertical stratification of the HR has been well documented in birds [9]; however, these studies quantified space use by comparing heights directly and ignored the other two dimensions, whereas others created 2D utilization distributions and then separately analysed vertical data. Regrettably, by assuming that individuals utilize the same vertical space, traditional 2D spatial analyses computed through the horizontal plane can likely overestimate the overlap between individuals that stratify vertically [10].

To date, very few studies examined the multidimensional space use of animals, arguably because of the lack of tools adapted to 3D data. Recently, however, two methods have been proposed to estimate 3D HRs. A study by [8] first stressed the inadequacy of existing modelling techniques to draw advantages from three-dimensional datasets and proposed the use of 3D kernel density estimators [11] to calculate 3D probabilistic HRs (i.e., *x*, *y*, *z* and *p*; where *p* measures the probability density that an animal is found at a given point within a certain 3D space). A study by [12] first proposed a 3D volumetric (i.e., *x*, *y* and *h_g_*; where *h_g_* is the height above ground level) HR estimator based on initial 2D HR estimation, followed by square (or hexagon) tessellation and 3D extrusion that assembled the 3D volumetric HR in the form of *n* adjacent parallelepipeds with different heights above the topographic surface.

If the issue of calculating 3D HRs has been adequately approached to date, the same is not true for their overlap. As yet, only studies concerning the pairwise overlap of 3D utilization distributions (i.e., 3D probabilistic HRs) have been proposed. A study by [13] first suggested a methodology to estimate the pairwise (i.e., *n* = 2) overlap between 3D probabilistic HRs by the adaptation of 2D overlap indices. A study by [14] solved the issue of computing the non-pairwise overlap of an arbitrary number (i.e., *n* ≥ 2) of probabilistic HRs for the first time, but the solution was limited to 2D utilization distributions (i.e., *x*, *y* and *p*; where *p* measures the probability density that an animal is found at a given point within a certain 2D space). Although 2D probabilistic HRs can be considered as a particular case of 3D volumetric HRs (HR_3D_ hereafter), where *z* = *p*, they pose two strong simplifications for overlap estimation when compared with volumetric ones. Firstly, in 2D probabilistic HRs the sum of *p* values (that corresponds to the volume under the probabilistic surface) is equal to 1 (or 100%) by definition [15]. By contrast, in the HR_3D_ the volume is measured in cubic kilometres and can assume any possible value ≥ 0 [12]. Secondly, in the 2D probabilistic HRs, the lower reference surface is simply a two-dimensional plane (*x*, *y*) where animal locations are placed, which can be operatively considered as a flat surface at *z* = 0 m a.s.l. Instead, HR_3D_ estimation raise the challenging issue of taking into account the topographic surface over which the HR_3D_ lays. In fact, the lower reference surface here is a (sometimes very complex) topography where each pair of coordinates is associated to a particular topographic height a.s.l. that can assume any possible value, even ≤0. These differences make the index developed for multiple overlap of 2D probabilistic HRs [14] useless for the HR_3D_.

Accordingly, in this study we solved the issue of measuring the degree of overlap/segregation among an arbitrarily large number (i.e., *n* ≥ 2) of HR_3D_ for the first time. To this aim, we upgraded the general overlap index [16] that was recently introduced for the computation of non-pairwise 2D HR overlap among *n* individuals, populations or species. Using spatial location data from the lesser kestrels *Falco naumanni* in southern Italy, we demonstrated the properties of our new index and discussed its potential applications to behavioural and movement ecology.

## 2. Materials and Methods

### 2.1. Study Species and Study Area

**Figure 1:**
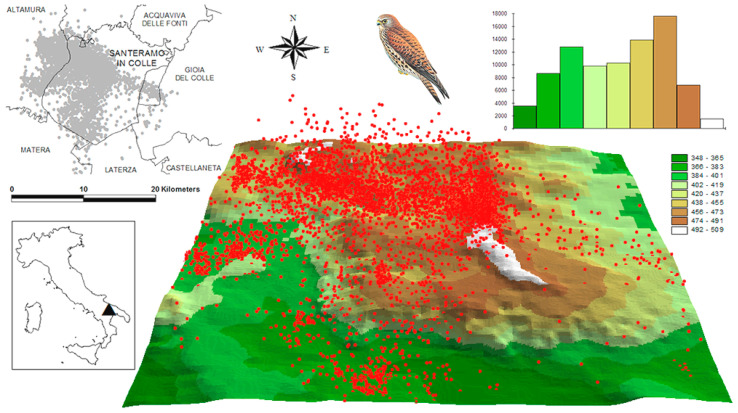
Study system. Top left: 2D map of the study area and GPS data (12,081 points in grey). Top right: elevation map histogram (X-axis: 9 equal-interval categories; Y-axis: number of GIS pixels; 1 pixel = 50 m × 50 m) of the study area. Centre: 3D representation of the topographic surface and GPS data (in red).

The study species is a small raptor present among the Annex I species of EU Wild Birds Directive 2009/147/EEC. This species relies on natural cliffs or rural and urban buildings as nesting sites and breeds in steppe-like grasslands and non-irrigated arable crops where it forages predominantly on invertebrates (in particular, grasshoppers [17]). The study area corresponded to the lesser kestrel colony of Santeramo in Colle (Apulia region, southern Italy; Figure 1), i.e., an agricultural landscape with an elevation gradient from 348 to 509 m a.s.l., located within the SPA (Special Protection Area) “Murgia Alta” IT9120007 and included within the Important Bird Area “Murge”. This area has the most elevated density of lesser kestrels in urban colonies worldwide [18]. The lesser kestrel population of Santeramo in Colle is characterized by an elevated within-colony overlap for both 2D [16] and probabilistic [14] HRs and elevated segregation with the adjacent lesser kestrel colonies [16].

### 2.2. Tagging of Birds

We tracked five lesser kestrels between 13 and 29 June 2017 during the nesting period (Table 1). We fitted the lesser kestrels with data loggers at their nest boxes. We employed TechnoSmart GiPSy-4 and GiPSy-5 data loggers (23 mm × 15 mm × 6 mm, <5 g weight) in order to collect spatio-temporal information (date, time, latitude, longitude, height a.s.l. and speed). The weight of the loggers in relation to that of the tracked individuals was <4%. All devices were tied dorsally using a 2 mm large Teflon tape knotted with a triple knot, and two tapes were crossed without a knot at the height of the sternum. We downloaded the data from the data loggers after the birds were recaptured at their nest boxes. Data acquisition occurred every three minutes following deployment.

### 2.3. Data Preparation

About 93% of the GPS points (i.e., 12,081 out of 12,984 points) were linked to 6–8 satellites. In these favourable conditions, the error associated to the measurement of the height a.s.l. (*h_s_*) was in the order of ±4–6 m [19]. We discarded the remaining 903 GPS points, linked to 5 or fewer satellites, because the measurement error associated to *h_s_* was in the order of ±20–25 m [19]. For each individual we also excluded the GPS points with *h_s_* greater than the 95th percentile (critical threshold) of the frequency distribution, so as to remove vertical outliers (possibly due to anomalous fine-scale weather conditions or GPS issues, e.g., atmospheric interference, low battery, multi-path effects) that could have determined an overestimation of the HR_3D_. GPS data were transferred to the ArcView GIS [20] and superimposed on the topographic surface of the study area (Figure 1) digitized at a 1:2000 scale by the authors from the available topographic maps of Apulia Region. For each GPS point, the height above ground level (in meters) was calculated as

(1) $$h_{g}=h_{s}-h_{t}$$

where *h_t_* is topographic height a.s.l.

In order to make pairwise comparisons between the statistical distributions of bird heights above ground level, we used a two-sample z-test. Given the 5 individuals, we performed 5 × (5 − 1)/2 = 10 pairwise tests that were considered to be significant for (two sided) *p* < 0.05.

### 2.4. 3D Volumetric Home Ranges

Within GIS, we calculated the HR_3D_ by using the methodology developed by [12]. Firstly, we computed the 2D polygonal HR of each individual. We employed the minimum convex polygon algorithm [21] with fixed arithmetic mean of all *x* (longitude) and *y* (latitude) coordinates and retained 95% of points closest to the arithmetic mean point. The 95% isopleth is widely used in the literature to exclude possible horizontal (i.e., in the *X*–*Y* plane) outliers due to fine-scale weather conditions or errors affecting GPS accuracy [22]. Secondly, we computed the smallest possible convex polygon (*SCP*) around the detected 2D HRs so as to delimit the study area over which the HR_3D_ and overlaps had to be calculated. Thirdly, we tessellated the *SCP* by using a regular square grid where the square size was determined through a trial-and-error computation. We used a set of possible candidates ranging from 1 hectare (i.e., 0.01 km^2^) to *SCP* incremented by 0.1 hectares, and the square size corresponded to the minimum size so that at least 70% of the squares contained at least 1 GPS point inside (i.e., *k* ≥ 1).

Fourthly, for each individual we assigned the maximum value of *h_g_* (i.e., *h_g_*^max^) to each square. In case of squares with no GPS points inside, we set *h_g_*^max^ = 0 m a.g.l. In mathematical terms,

(2) $$\left\{ \begin{array}{l} h_{g}{}^{\max}=\max h_{g}\;\mathrm{if}\;k\geq 1\\ h_{g}{}^{\max}=0\;\mathrm{if}\;k=0 \end{array}\right.$$

Fifthly, for each individual we extruded each square upon the topographic surface by an elevation equal to *h_g_*^max^. This step assembled a polyhedron in the form of *n* adjacent parallelepipeds above the topographic surface, therefore the HR_3D_ of each lesser kestrel was the solid figure bounded superiorly by the upper surface of the polyhedron and inferiorly by the topographic surface.

Lastly, for each lesser kestrel, the volume of the HR_3D_ was calculated as

(3) $$V_{3D}=\sum_{\;\mathrm{SCP}}\sum h_{g}{}^{\max}\Delta x\Delta y$$

or

(4) $$V_{3D}=\iint_{SCP}h_{g}{}^{\max}dxdy$$

in case Δx ≅ 0 and Δy ≅ 0. Although the 2D HR of each lesser kestrel was a sub-region of the *SCP*, we used the *SCP* as the domain of Equations (3) and (4) because *h_g_*^max^ was equal to 0 outside the 2D HR, therefore *V_3D_* assumed the same value over these two domains.

### 2.5. 3D Home Range Overlap

We upgraded the recently introduced general overlap index (*GOI*; [16]) that allows for the computation of non-pairwise overlap among an arbitrarily large number (*n* ≥ 2) of 2D HRs. The *GOI* follows a simple idea: given *n* 2D HRs, it is always possible to calculate the extent of two spatial configurations, perfect segregation and perfect overlap. The *GOI* simply measures the distance of the observed overlaps from these two extremes. In the case of perfectly disjointed (i.e., perfect segregation) 2D HRs, the total area (*T_A_*) covered by the 2D HRs is simply the sum of their extents $\sum A_{i}$. In the case of perfectly nested (i.e., perfect overlap) 2D HRs, *T_A_* is the extent of the largest HR (i.e., *max*(*A_i_*)). In the intermediate case (i.e., partially overlapping HRs), *T_A_* corresponds to the spatial union of the HR polygons, $\cup A_{i}$. The larger the overlap, the smaller the $\cup A_{i}$. The *GOI* is simply the ratio between the observed (*Dist_OBS_*) and maximum (*Dist_MAX_*) distances from the perfectly segregated (i.e., non-overlapping) situation, calculated as

(5) $$GOI=100\times \frac{Dist_{OBS}}{Dist_{MAX}}=100\times \frac{\sum_{i=1}^{n}A_{i}-\overset{n}{\underset{i=1}{\cup }}A_{i}}{\sum_{i=1}^{n}A_{i}-\max(A_{i})}$$

If *Dist_OBS_* = 0 (i.e., perfect non-overlap), then the *GOI* = 0; if *Dist_OBS_* = *Dist_MAX_* (i.e., perfect overlap), then the *GOI* = 100. In the intermediate cases, then 0 < *GOI* < 100. A general segregation index (*GSI*) can be computed as the complement to 100 of the *GOI*. A geometrical elucidation of these two indices can be found in [16].

Both the *GOI* and *GSI* only consider the 2D spatial domain of the individual HRs and ignore the third dimension (i.e., *h_s_*, *h_g_* and *h_t_*) associated to each GPS points. In terms of volumetric HRs, in case of perfect segregation $\sum A_{i}$ becomes the sum of the HR_3D_.

(6) $$\sum_{i=1}^{n}V_{3D_{i}}={\sum_{i=1}^{n}\left(\underset{\;\mathrm{SCP}}{\sum \sum }h_{g}{}^{\max}\Delta x\Delta y\right)}_{i}$$

In case of perfect overlap, *max*(*A_i_*) simply becomes the largest HR_3D_

(7) $$\max(V_{3D_{i}})=\max{\left(\underset{\;\mathrm{SCP}}{\sum \sum }h_{g}{}^{\max}\Delta x\Delta y\right)}_{i}$$

In the intermediate case (partially overlapping HR3_D_), $\cup A_{i}$ corresponds to the spatial union of the HR_3D_, i.e., the volumetric HR where each grid square assumes the maximum value among all the *h_g_*^max^ of the HR_3D_, calculated as

(8) $$\overset{n}{\underset{i=1}{\cup }}V_{3D_{i}}=\underset{\;\mathrm{SCP}}{\sum \sum }\max{\left(h_{g}{}^{\max}\right)}_{i}\Delta x\Delta y$$

By inserting Equations (6)–(8) into Equation (5), the non-pairwise overlap among an arbitrarily large number (*n* ≥ 2) of HR_3D_ reads as

(9) $$MVOI=100\times \frac{{\overset{n}{\underset{i=1}{\sum }}\left(\underset{\;\mathrm{SCP}}{\sum \sum }h_{g}{}^{\max}\Delta x\Delta y\right)}_{i}-\underset{\;\mathrm{SCP}}{\sum \sum }\max{\left(h_{g}{}^{\max}\right)}_{i}\Delta x\Delta y}{{\sum_{i=1}^{n}\left(\underset{\;\mathrm{SCP}}{\sum \sum }h_{g}{}^{\max}\Delta x\Delta y\right)}_{i}-\max{\left(\underset{\;\mathrm{SCP}}{\sum \sum }h_{g}{}^{\max}\Delta x\Delta y\right)}_{i}}$$

In case Δx ≅ 0 and Δy ≅ 0, the *MVOI* must be calculated by using double integrals as

(10) $$MVOI=100\times \frac{{\sum_{i=1}^{n}\left(\iint_{SCP}h_{g}{}^{\max}dxdy\right)}_{i}-\iint_{SCP}\max{\left(h_{g}{}^{\max}\right)}_{i}dxdy}{{\sum_{i=1}^{n}\left(\iint_{SCP}h_{g}{}^{\max}dxdy\right)}_{i}-\max{\left(\iint_{SCP}h_{g}{}^{\max}dxdy\right)}_{i}}$$

The *MVOI* can also be calculated by directly using *h_s_* and *h_t_* as follows

(11) $$MVOI=100\times \frac{{\sum_{i=1}^{n}\left(\iint_{SCP}\int_{h_{t}}^{h_{t}+h_{g}{}^{\max}}h_{s}\;dxdydz\right)}_{i}-\iint_{SCP}\int_{h_{t}}^{h_{t}+h_{g}{}^{\max}}\max{\left(h_{s}\right)}_{i}\;dxdydz}{{\sum_{i=1}^{n}\left(\iint_{SCP}\int_{h_{t}}^{h_{t}+h_{g}{}^{\max}}h_{s}\;dxdydz\right)}_{i}-\max{\left(\iint_{SCP}\int_{h_{t}}^{h_{t}+h_{g}{}^{\max}}h_{s}\;dxdydz\right)}_{i}}$$

Equations (10) and (11) return the same result, but Equation (10) is probably more straightforward. Finally, a volumetric general segregation index (*MVSI*) can be computed as

(12) MVSI=100%−MVOI

## 3. Results

**Figure 2:**
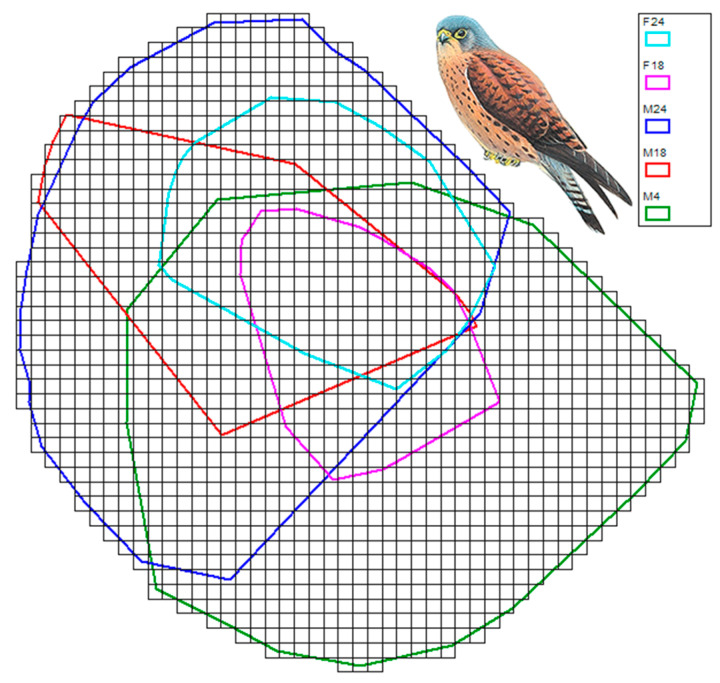
Tessellation (1584 squares) of the smallest possible convex polygon around the 2D home ranges (coloured polygons) of the tracked lesser kestrels. IDs are the same as those in Table 1.

The five 2D HRs ranged from 22.76 to 111.48 km^2^, with lower values for the female lesser kestrels. The *SCP* was 142.4 km^2^ (Figure 2). After several trial-and-error attempts, the square size was set to 0.09 km^2^ (i.e., Δ*x* = Δ*y* = 300 m); therefore, the *SCP* was covered with 1584 squares.

**Figure 3:**
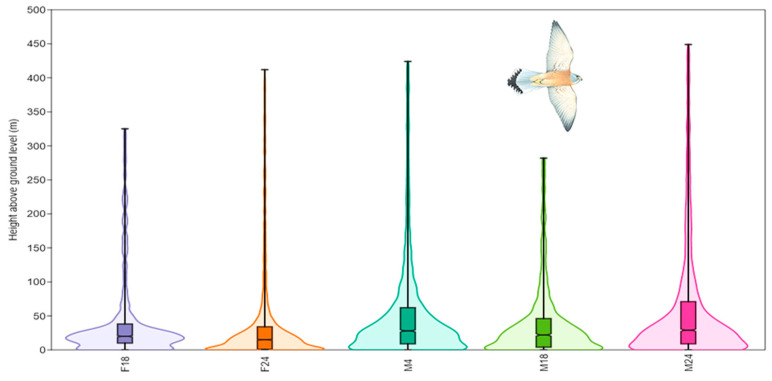
Violin plots showing the probability density of the heights above ground level (Y-axis) of the GPS points of each lesser kestrel. The box plots inside the violin plots show median (horizontal line inside the box) and the 25–75 percent quartiles. IDs are the same as those in Table 1.

The tracked lesser kestrels showed dissimilar vertical profiles (Figure 3). All the pairwise differences between the statistical distributions of bird heights above ground level were significant, except for the two pairs of individuals F18–M18 and F24–M18 (Table 2).

The median *h_g_* of the tracked individuals ranged from 16 to 29 m a.g.l., while the 95th percentiles of *h_g_* were between 283 and 448 m a.g.l. (Table 3). The female F18 presented many GPS points with *h_g_ >* 150 m and up to 250 m a.g.l., though the most frequent *h_g_* were those around the median (20 m a.g.l.). The female F24 preferred lower *h_g_* (the 25th percentile of *h_g_* was close to 0 m a.g.l.), and the probability density of *h_g_* decreased constantly as *h_g_* increased and was almost null for *h_g_* > 100 m a.g.l. The male M4 showed two peaks of the probability density of *h_g_*, the first peak close to 0 m a.g.l. and the second peak close to the median (29 m a.g.l.). In addition, it showed an elevated amount of GPS points up to 200 m a.g.l. The male lesser kestrel M18 preferred lower *h_g_* (the 25th percentile of *h_g_* was close to 0 m a.g.l.), but it showed a fair amount of GPS points up to 200 m a.g.l. The male lesser kestrel M24 exhibited the highest values of *h_g_* for both the 75th (71 m a.g.l.) and 95th percentile (448 m a.g.l.).

**Figure 4:**
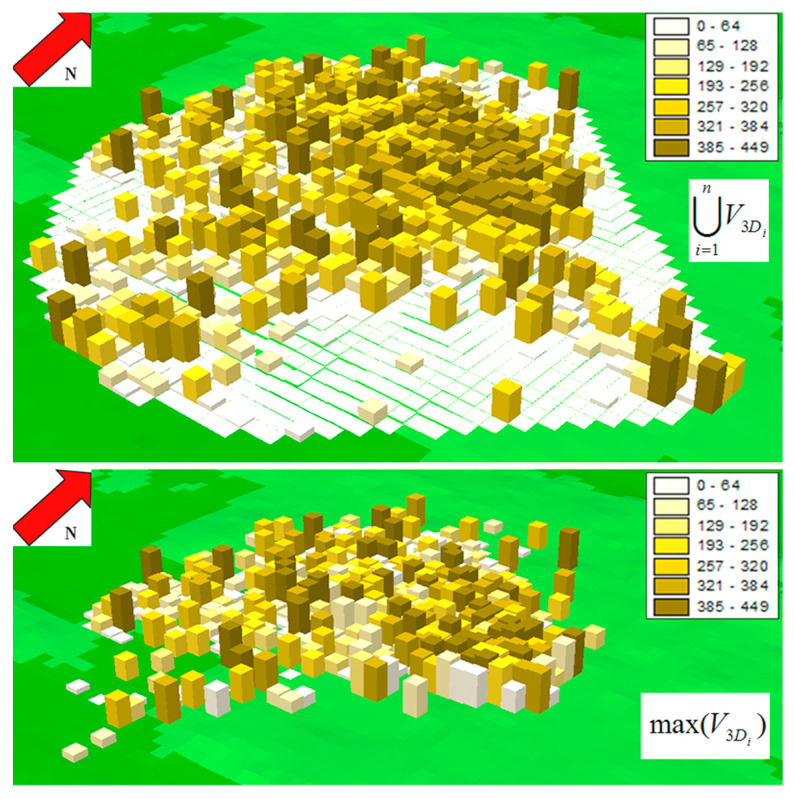
Top: spatial union of the 3D volumetric home ranges of the tracked lesser kestrels (volume = 15.08 km^3^). Bottom: largest 3D volumetric home range (lesser kestrel ID M24; volume = 8.19 km^3^). The legends show heights above ground level (in meters) of the parallelepipeds constructed by extruding the 1584 squares of Figure 2.

The five HR_3D_s ranged between 1.79 km^3^ and 8.19 km^3^ (Table 3). *Max(V_3D_)* and *ΣV_3D_* were 8.19 km^3^ and 21.89 km^3^, respectively. The volume of $\cup V_{3D}$ was 15.08 km^3^ (Figure 4), thus the *MVOI* was 100 × (21.89 − 15.08)/(21.89 − 8.19) = 49.71% and the *MVSI* was 100% − 49.71% = 50.29%.

## 4. Discussion

The issue of estimating the degree of overlap among multiple 3D volumetric HRs has remained unsolved to date. Accordingly, in this study we first introduced a non-pairwise metric of overlap/segregation among multiple HR_3D_, whose ecological interpretation is much easier and more meaningful if compared to *n* × *n* pairwise overlap matrices computed through pairwise indices. In addition, one overlap index is more effective if estimates of the overlap are to be meaningfully compared across several studies. This fulfils the demand by many authors [23,24] who argued that any overlap index should be intuitive and easy to interpret.

Incorporating the vertical component into the representations of animal space use can provide novel ecological insights with benefits for conservation management [8]. Although 2D analyses are informative about the locations of the tracked individuals, volumetric analyses provide the benefit of combining 3D information into metrics that represent the actual space use of animals [10]. In fact, when animal behaviour includes movements with a substantial vertical component, like in birds, simplifying the assumptions of 2D HRs can affect ecological inferences by overestimating interactions between individuals, population and/or species [25]. By contrast, three-dimensional spatial analyses enable the accurate description of the patterns of spatial overlap/segregation [10]. Results from our analyses support this argument based on the increased amount of detailed information gained from incorporating the vertical dimension with animal location information. In fact, in our previous studies we found that the non-pairwise overlap index (*GOI*) among the 2D HRs of the lesser kestrels used in this study was 81.38% [16], and the overlap was 94.016% by using a non-pairwise probabilistic HR index (*PGOI*; probabilistic general overlap index; [14]). In both cases, the degree of overlap among the individuals of this colony was very elevated. In this study, we found that their 3D HR overlap was only 61.1% of the 2D overlap and 52.8% of the probabilistic one. This difference in the estimation of overlap occurred because the 3D analysis allowed separation between individuals that occurred in the same location but showed different vertical profiles above ground level. The lesser kestrel colonies present in the study area (e.g., Altamura, Cassano Murge, Gravina, Santeramo in Colle) are characterized by elevated segregation with the adjacent colonies [16,26]. Because the colony-specific home ranges result in mutually exclusive areas, each colony has only limited space available, which raises intra-colony competition due to increased 2D overlap among individuals. Accordingly, we hypothesize that the detected segregation along the vertical dimension could be an adaptive behaviour employed by the lesser kestrel population of this colony to decrease the elevated intra-colony competition. Alternatively put, we suggest that, as a general rule, the larger the intra-colony 2D overlap is, the larger the 3D segregation will be.

We argue that these three non-pairwise metrics of HR overlap/segregation (i.e., *GOI*, *PGOI*, *MVOI*) quantify the different properties of the animal space use, thus none is exhaustive if considered separately, and instead they should be combined into a vector of three synthetic metrics capable of taking into account all the fundamental properties of animal HR overlap/segregation.

### 4.1. Applications to Tracking Studies

The methodological approach proposed here enabled the description of animal distribution in the same number of dimensions as the environment in which they live, thus providing a realistic description of their space use. The most basic and intuitive application of our HR_3D_ overlap estimator is to gain ecological insights into the volumetric requirements of birds in order to endure in their distribution areas. For example, the smallest HR_3D_ found in this study (i.e., female lesser kestrel ID F18; HR_3D_ = 1.79 km^3^) involved a 3D space above ground level equal to: (a) 716,000 Olympic-sized swimming pools (i.e., 50 m × 25 m × 2 m), (b) 1356 Colosseums or (c) 737 Great Pyramids of Giza. Instead, the 3D HR overlap indicates where most birds can find the most suitable conditions in the 3D environment, which includes (a) low disturbance due to anthropogenic structures with considerable vertical dimension (e.g., wind farms and power lines [27,28]); (b) favourable vertical thermal gradients (bands of warm and cool air) and air currents (headwinds, tailwinds, crosswinds); and (c) non-negative interactions with other avian species. The application of 3D analyses to the study of biotic interactions could provide the ability to better understand these interactions over current 2D approaches. For example, in the study area, the lesser kestrel is predated by the magpie (*Pica pica)*, the lanner falcon (*Falco biarmicus*) and the peregrine falcon (*Falco peregrinus*) that occupy the same 2D space as that of lesser kestrels [29]. The application of our overlap/segregation metrics to the HR_3D_ of these species could possibly reveal that these predators have different degrees of 3D overlap with the lesser kestrel, and thus the different degree of biotic interactions. Overall, our results suggest that airspace is a habitat with as much potential to influence birds’ movements and space use as other environments, and it should not be considered only as something birds move through between land or water habitats.

Although the basic utilization of the *MVOI* and *MVSI* deals with 3D space use and overlap at individual, population or species level, we suggest two further possible applications. Time has rarely been considered when estimating HR size and overlap [30], and it has never been incorporated as a fourth dimension (i.e., 4D volumetric home ranges; x, y, *h_g_* and t). Space use can vary in time and such variations may have implications for habitat selection and use, trophic interactions and predator–prey relationships [9]. Accordingly, the *MVOI* and *MVSI* can be used to quantify how the individuals’ HR_3D_s overlap changes over time in a certain population (e.g., between life history stages or before and after experimental manipulations) or to evaluate the degree of overlap between the HR_3D_ of the same individual at different phenological stages (e.g., nesting and post-nesting periods). Another important application of our new metrics is the evaluation of the robustness of the HR assessment through the degree of overlap of several HR_3D_ estimators: if they are in good agreement, then the *MVOI* will be close to 100.

By combining overlaps into a single metric, one could lose useful information regarding the extent of overlap between animals with different attributes (e.g., sex, size, diet preference, nest location, etc.), from which one could obtain statistics. Accordingly, although we conceived the *MVOI* and *MVSI* as non-pairwise metrics among multiple 3D home ranges, nothing prevents researchers from applying them in a pairwise manner by splitting the dataset of the tracked animals based on the attribute of interest (e.g., individual *i* versus individual *j*, females versus males, youngs versus adults etc.).

### 4.2. Mathematical Properties of the Proposed Metrics

The proposed metrics have the same mathematical properties as the *GOI* and the *PGOI*: (1) whatever the number of HR_3D_s under study, the *MVOI* and *MVSI* return a single overlap measure; (2) in case of perfectly segregated HR_3D_, the *MVOI* is equal to 0 and the *MVSI* to 100; (3) in case of perfectly overlapping HR_3D_, the *MVOI* is equal to 100 and the *MVSI* to 0; and (4) in any other case, the *MVOI* and *MVSI* return a value between 0 and 100. Although apparently complex, the *MVOI* corresponds to the linear equation Y = 100 × (b − X)/(b − a), where *a* is the volume of the largest HR_3D_, *b* is the sum of the HR_3D_ and *X* is the volume of the union of the HR_3D_, which varies depending upon the degree of overlap. The first derivative of the *MVOI* with respect to *X* is equal to −100/(*b* − *a*), thus every unitary (e.g., 1 km^3^) increase/decrease of $\cup V_{3D}$ (due, for instance, to changes of the HR_3D_ over time) determines a decrease/increase in the *MVOI* that is constant and independent of the initial value assumed by $\cup V_{3D}$. This assures that small/big changes to the HR_3D_ overlaps proportionally determine small/big changes to our overlap indices, regardless of the initial degree of overlap.

## 5. Conclusions

While it is evident that animals live in a 3D environment, to date, biotelemetry data have mostly been analysed and modelled in two dimensions. However, advances in biotelemetry are increasingly providing the opportunity to gather additional data beyond 2D location coordinates.

Accordingly, in this study, we proposed a new approach that integrates mathematical concepts with telemetry data to provide the opportunity to define 3D space overlap among *n* individuals, populations or species. The usefulness of our metrics is not limited to avian studies and can be applied to any dataset that includes 3D coordinates. A further strong point of our new metrics is that they can be calculated using standard GIS operations and can be obtained by using any free GIS software.

## Figures and Tables

**Table 1:** Data of the GPS-tracked lesser kestrels.

GPS	Sex	Weight	Start Date	End Date	Number of
ID		(g)	of Tracking	of Tracking	GPS Points
F18	F	155	13 June 2017	16 June 2017	1375
F24	F	126	22 June 2017	29 June 2017	3311
M4	M	128	16 June 2017	22 June 2017	2765
M18	M	135	13 June 2017	16 June 2017	1417
M24	M	126	22 June 2017	29 June 2017	3213

**Table 2:** Pairwise comparisons between the statistical distributions of bird heights above ground level by using a two-sample z-test. The table shows the pairwise z-values along with statistical significance. Tests were considered to be significant for (two sided) *p* < 0.05.

Z-Statistics	F18	F24	M4	M18	M24
F18		−2.33 *	4.12 **	−1.41	7.33 **
F24			7.60 **	0.92	11.24 **
M4				−6.11 **	3.68 **
M18					9.55 **
M24					

* *p* < 0.05, ** *p* < 0.01.

**Table 3:** For each lesser kestrel, the 2D and 3D properties of space use are shown. a.g.l. stands for ‘above ground level’. IDs are the same as those in Table 1.

GPS ID	2D Home Range	Average Height	Median Height	95th Percentile	3D Home Range
	(km^2^)	a.g.l. (m)	a.g.l. (m)	height a.g.l. (m)	a.g.l. (km^3^)
F18	22.76	44	20	325	1.79
F24	34.32	39	16	412	4.46
M4	111.48	54	29	424	5.58
M18	40.46	40	21	283	1.87
M24	97.62	62	29	448	8.19

## Data Availability

The dataset used in this study is available from the first author on reasonable request.
